# The German R&D Program for CO_2_ Utilization—Innovations for a Green Economy

**DOI:** 10.1007/s11356-016-6641-1

**Published:** 2016-04-20

**Authors:** Lothar Mennicken, Alexander Janz, Stefanie Roth

**Affiliations:** Division Resources and Sustainability, Federal Ministry of Education and Research, Bonn, Germany; Forschungszentrum Jülich GmbH, Project Management Jülich, Berlin, Germany

**Keywords:** Carbon capture and utilization, CO2 utilization, CO2 mitigation, Power-to-X, Government policy, Germany, Sustainability, Research, development, and innovation

## Abstract

Carbon capture and utilization (CCU) is a field of key emerging technologies. CCU can support the economy to decrease the dependency on fossil carbon raw materials, to stabilize electricity grids and markets with respect to a growing share of fluctuating renewable energy. Furthermore, it can contribute to mitigate anthropogenic CO_2_ emissions. The German Federal Ministry of Education and Research has provided substantial financial support for research and development projects, stimulating research, development, and innovations in the field of CO_2_ utilization. This review provides an overview over the most relevant funding measures in this field. Examples of successful projects demonstrate that CCU technologies are already economically viable or technologically ready for industrial application. CCU technologies as elements of a future “green economy” can contribute to reach the ambitious German sustainability targets with regard to climate protection as well as raw material productivity.

## Introduction

Germany is fully aware of the challenges of the climate change. Therefore, the Federal Government has expressed the national climate goals within the “National Sustainability Strategy” in 2002 (Germany 2002). Germany has set ambitious targets: to reduce greenhouse gas emissions by 40 % by 2020 as compared to 1990 and to 80 % by 2050, respectively. Also, the raw material productivity is to be doubled until 2020. To meet these targets, Germany defined the “High Tech Strategy”, which was renewed in 2015 as the “New High-Tech-Strategy” (BMBF 2014b). The New High-Tech Strategy defines different top priorities for the future. Raw material efficiency is a substantial contributor to the field of “sustainable economy and energy.”

As an executional measure for the New German High-Tech Strategy, the Federal Ministry of Education and Research (Bundesministerium für Bildung und Forschung, BMBF) has announced the major research framework program “FONA^3^—research for sustainable development.” In its third edition, FONA has undergone some major changes. Now, three so-called flagship initiatives—green economy, future cities, and energiewende (energy transition)—have been announced and they are accompanied by three main research areas. Here, the intelligent use of resources is a topic of priority (BMBF 2015d).

The intelligent use of resources is of specific importance for the German chemical industry. The chemical industry supplies automotive industry as well as pharma and cosmetic corporations and thus is on the base of the German economy and its growth. Due to the fact that Germany today strongly depends on the import of fossil raw materials for non-energy uses, it is of strategic interest to sustainably broaden the raw material base of the chemical industry. New technologies and processes could lead to the use of CO_2_ as a raw material. Consequently, fossil resources could be substituted, higher resource efficiency could be achieved, and CO_2_ emissions could be mitigated to a certain extent.

This review provides an overview over the public funding of research and development (R&D) in Germany by the BMBF with respect to CO_2_ utilization. Additionally, selected projects will be presented.

## BMBF funding measures in CO_2_ utilization

The Federal Ministry of Education and Research (BMBF) funds a broad variety of R&D projects in various programs. Most funding measures with regard to carbon dioxide capture and utilization (CCU) are clustered within the research framework program FONA^3^.

### Technologies for Sustainability and Climate Protection: Chemical Processes and Use of CO_2_

In May 2009, Germany initiated as one of the first nations in the world a major research program in CCU: “Technologies for Sustainability and Climate Protection: Chemical Processes and Use of CO_2_” (BMBF 2009). Between 2010 and 2016, approximately 100 million Euros have been granted for 33 collaborative research and development projects, consisting of more than 150 individual projects. In addition, the industrial partners invested further 50 million Euros to support the research projects. With this, the funding measure belongs worldwide to the largest, specifically targeted governmental support programs for CCU.

The funding measure focused on the following major research goals:

- Extension of the raw material base of the chemical industry by utilizing CO_2_ for the synthesis of basic chemicals
- Utilization of CO_2_ for chemical energy storage
- Chemical activation of CO_2_ by the means of catalysis
- Innovations in CO_2_ capture and separation, e.g., from flue gas
- Reduction of greenhouse gas emissions in energy-intensive processes by increased energy efficiency and the use of functional solvents

Additionally, three academic junior research groups are supported within this funding activity: (I) Dr Mayrhofer investigates a project to develop novel high-throughput methods for electrocatalysts with a specific focus on direct CO_2_ reduction. For his outstanding work, he was awarded the Science Award Electrochemistry in 2013 and the DECHEMA Prize in 2014 (BASF 2013; DECHEMA 2014). (II) Dr Strunk works successfully on the development of active and selective heterogeneous photocatalysts for the reduction of CO_2_ to C1 building blocks. She received the Jochen Block Award of the German Catalysis Society in 2014. (III) The objective of Dr Werner’s research is the development of new organocatalysts for the utilization of CO_2_ as a building block for chemical synthesis.

To interlink the projects, DECHEMA (Gesellschaft für Chemische Technik und Biotechnologie e.V., Society for Chemical Engineering and Biotechnology) coordinates a support and transfer project. The carbon footprint and economic potential of the new technologies will be evaluated.

Many of the projects were highly successful and can be regarded as strong indicators for the great potential of CO_2_ utilization. In the following sections, three projects will be highlighted with respect to the major research goals: CO_2_ in polymers, energy storage, and energy efficiency.

#### CO_2_ as a raw material in polymer synthesis—Dream Production

The project team of “Dream Production,” led by Covestro AG in Leverkusen (formerly Bayer MaterialScience AG), had the vision to incorporate CO_2_ in new polymeric materials. As target products polyurethanes (PUR) were selected. Conventionally, PUR are made from isocyanates and polyols. Researchers of RWTH Aachen University and Covestro investigated the catalyzed reaction of epoxides with CO_2_ to form polyether carbonate polyols. Especially double metal cyanides (DMC) as catalyst, as for instance dinuclear zinc-cobalt-complexes, and multifunctional alcohols as reaction starters lead to the desired products (Langanke et al. 2014).

**Fig. 1:**
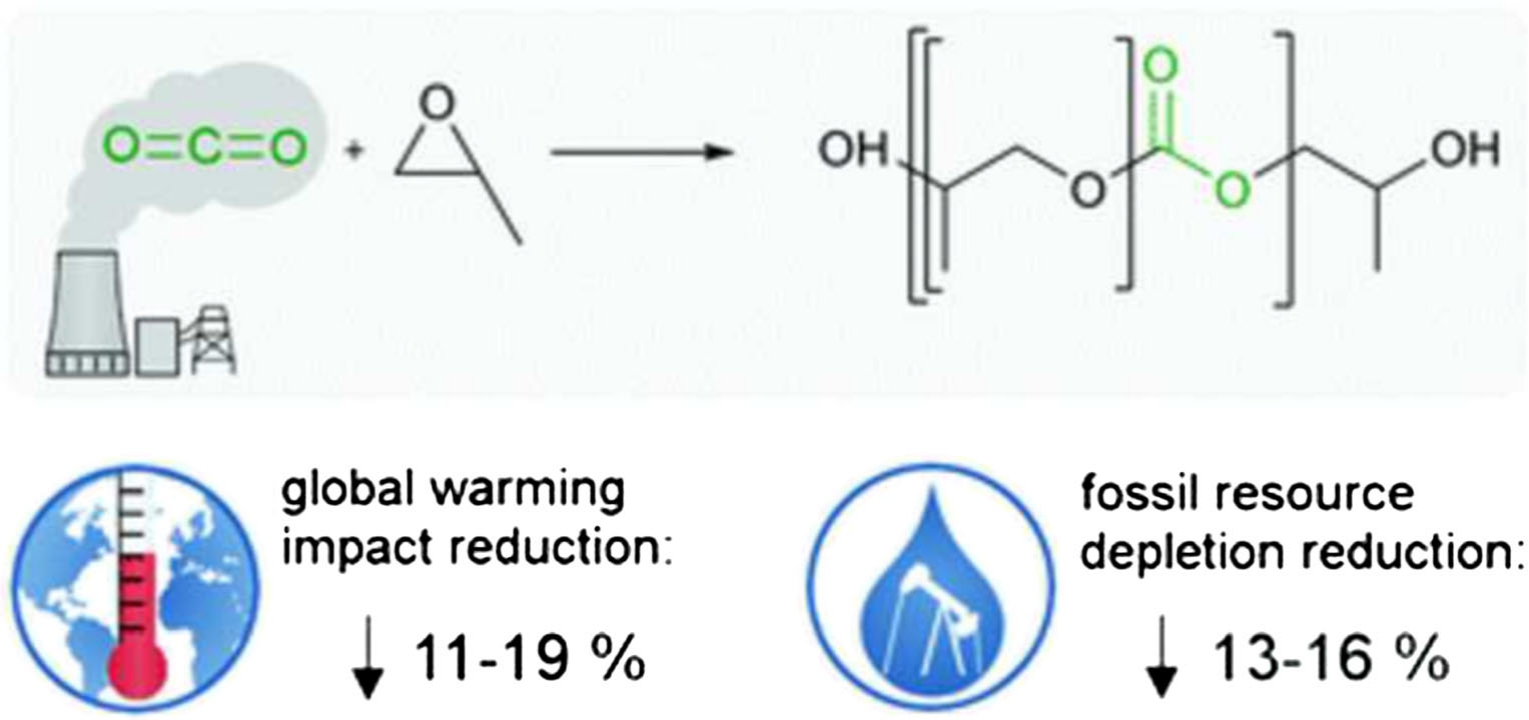
CO_2_ polyols of the Dream Production joint project and their environmental impact (von der Assen and Bardow 2014)

The aim of the project Dream Production was the scale-up of the laboratory results from the precursor project Dream Reaction to proof that a process in the technical scale is feasible with regard to economy and ecology. Therefore, researchers from RWTH Aachen University investigated the catalytic aspects of the conversion of power plant-derived CO_2_ and performed a comprehensive life cycle assessment (LCA; Fig. 1). For a realistic scenario, it was of great importance to obtain CO_2_ from an existing and large-scale point source, such as a power plant. Thus, the German energy supplier RWE Power became a partner of the Dream Production. RWE performed the separation of CO_2_ from a lignite-fired power plant. Covestro was responsible for the scale-up of the process. The results were striking: At 90 bar, CO_2_ was incorporated into polyether carbonate polyols with up to 22 wt%. In this composition, the polymer chains possess the optimal flexibility for the addressed PUR products. For comparison reasons, PUR was prepared with standard industrial equipment from polyether carbonate polyol with 10.5 wt% CO_2_. The resulting CO_2_-based PUR foam showed the same basic physical properties as conventional PU foams (Langanke et al. 2014).

Based on the results of Dream Production, Covestro currently builds the first commercial plant for a CO_2_-based polyurethane product at the Dormargen site in Germany, which is a mattress. In the middle of 2016, the first CO_2_-based mattresses are expected to be commercially available, e.g., the level of technology readiness (TRL) corresponds to 7–8. Results from the LCA indicate, as shown in Fig. 1, that the replacement of some of the fossil-based epoxides with CO_2_ lowers the CO_2_ emissions by approximately 20 % as compared to a conventional PUR foam product (von der Assen and Bardow 2014).

Further improvements in the replacement of fossil raw materials through the incorporation of CO_2_ were achieved in the project Dream Polymers. The new process incorporates CO_2_ twice: directly into a new kind of PUR precursor (polyoxymethylene polycarbonate polyol, POM PET) and indirectly by producing a precursor of POM PET. All together, up to 1.7 kg CO_2_ per kilogram PU can be utilized in the new process (von der Assen et al. 2015).

#### Synthetic fuels from CO_2_, water, and renewable energy substitute crude oil

The energy system in Germany is drastically changing towards renewable energy (Energiewende). Photovoltaic and wind power are, however, highly fluctuating, and energy storage becomes a major challenge. The production of hydrocarbons from “surplus” renewable energy has a strong potential to contribute to power grid stabilization, and the hydrocarbons can also easily be stored and distributed. Technologies that allow the transformation of renewable energy into hydrocarbons via water splitting are generally termed “Power-to-X,” with “X” being a placeholder for gas, liquids, or chemicals.

Hydrocarbons for the mobility sector will be indispensable for many decades. Short distance and public transportation might be transitioned into electromobility in the mid-term. Natural gas mobility is available already today with sufficient performances in private and public transportation as well as heavy goods transportation. Innovations in Power-to-Gas (PtG) technologies can increase the supply with sustainably generated synthetic natural gas (SNG).

For long-haul flights, heavy goods transportation, but also for cars, hydrocarbons will be demanded for many more decades. Power-to-Liquid technologies provide an access to sustainably produced hydrocarbons with a very low carbon footprint and might thus facilitate the replacement of fossil fuels for combustion engines in the future.

**Fig. 2:**
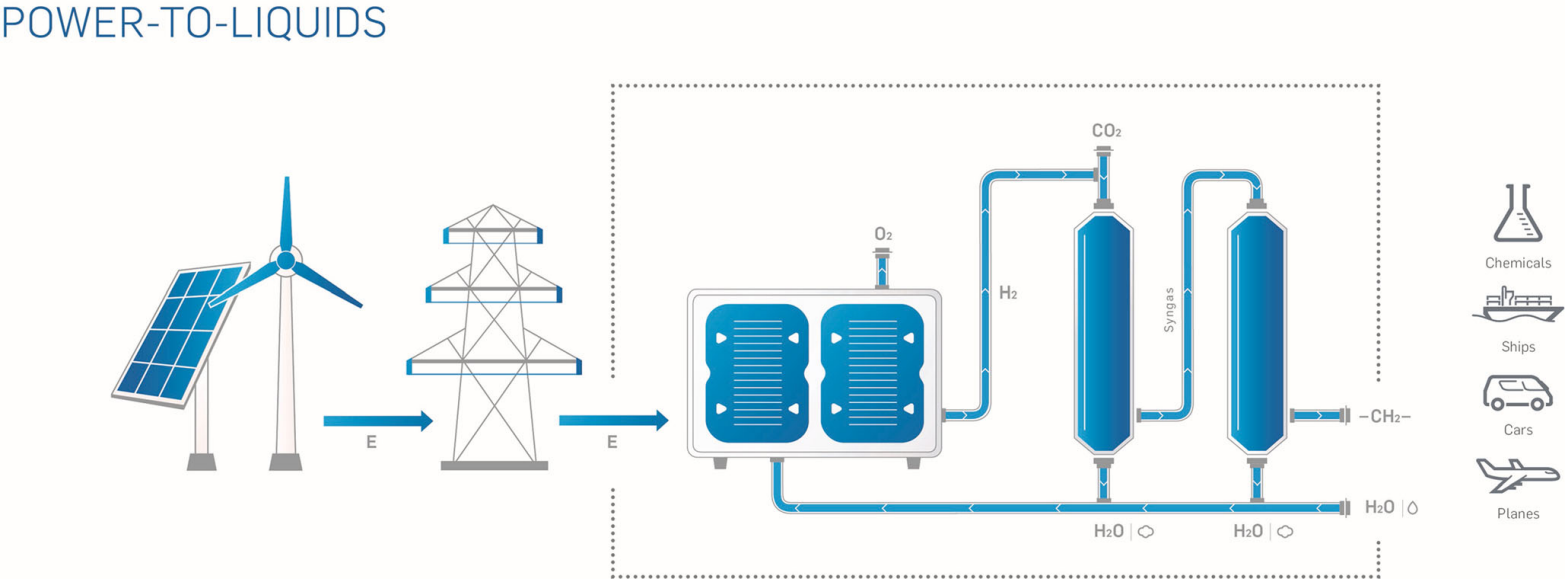
The sunfire process consists of high-temperature steam electrolysis, CO_2_ conversion via reverse water-gas shift reaction, a high temperature heat exchanger, and Fischer-Tropsch-synthesis (Sunfire 2015)

The Power-to-Liquid (PtL) technology, e.g., the “sunfire” technology developed by the Dresden based startup company sunfire, consists of three key elements, as displayed in Fig. 2: (1) high-temperature solid-oxide steam-electrolysis, (2) CO_2_ conversion via reverse water-gas shift reaction, and (3) Fischer-Tropsch synthesis. A first economic analysis based on numbers from 2014 was conducted. The electric energy costs for the production of synthetic fuels (plant and operating costs where not yet included) were estimated to 7 Eurocents per kilowatt hour. The total production costs would be much higher. Compared to the novel approach, fossil fuels were estimated to 6 Eurocents per kilowatt hour, based on crude oil price of $100 per barrel in 2014 (Verdegaal et al. 2015).

The high-temperature electrolyzer of the sunfire project reaches an electrical efficiency of more than 90 % from power to H_2_ by integrating waste heat from Fischer-Tropsch. It is designed to operate reversible as both, fuel-cell and electrolyzer, which creates a unique business model. The fuel cell/electrolyzer is able to produce high caloric chemicals, when renewable excess energy is available. In times of energy penury, the system supplies electricity by converting stored high caloric chemicals. Consequently, the novel technology creates a new income as operation reserve of the electrical grid (BMBF 2015f).

The three key elements electrolyzer/fuel cell, reverse water gas shift reaction, and Fischer-Tropsch reaction were combined and developed in an industrial relevant scale within the sunfire project. Based on the research results, the sunfire team designed and constructed a PtL demonstration plant that is worldwide the first of its kind. Currently, the plant is able to produce one barrel per day of synthetic diesel fuel from CO_2_, water, and renewable energy (von Olshausen and Rüger 2014). It is noteworthy that this synthetic liquid fuel is free of any sulfur, nitrogen, aromatic compounds, or fossil oil, and hence performs superior as compared to fossil fuels. Due to the Fischer-Tropsch unit, in addition to synthetic fuels, kerosene for aviation and waxes for the cosmetic industry can be produced by the sunfire technology (TRL 6).

In April 2015, the German Federal Minister of Education and Research, Johanna Wanka, fueled her official car with the first five liters of the so called “e-diesel”, refined from sunfire’s “Blue crude” for a test drive, which was recognized worldwide by global media coverage.

#### Energy efficiency—CO_2_ mitigation by nanofiltration

The chemical industry is by nature one of the highest energy-consuming industries. Significant reduction of CO_2_ emissions can only be achieved by key innovations in energy efficiency. A highly energy-demanding unit operation, used in the chemical industry, is the thermal separation of solvents. Novel, energy-efficient methods for the recovery of solvents can therefore significantly reduce CO_2_ emissions. Organophilic solvent nanofiltration (OSN), as investigated by the Evonik-led joint project “OPHINA,” is a promising technology for this purpose. The project team developed nanofiltration membranes with properties superior to commercially available membranes with regard to solvent stability, permeate flux, selectivity, and long-term mechanical stability. Compared to steam-based thermal separation processes, the cold separation technique OSN can save up to 92 % of CO_2_-eq. emissions (Schnitzer et al. 2014). After the end of the R&D project, Evonik developed commercial membranes for OSN applications, which are commercially available today (TRL 9).

### Twenty20—Partnership for Innovation

“Entrepreneurial Regions” is a BMBF Innovation Initiative for the New German Länder. Its focus lies on innovation-oriented regional alliances from academia and industry with common core competences. The R&D program “Twenty20—Partnership for Innovation” as one member of the program family “Entrepreneurial Regions” promotes the economic development of the former East German states by technology development (BMBF 2012). Until 2020 the BMBF grants up to 500 million Euros for nine consortia.

One of the nine granted concepts is HYPOS—Hydrogen Power Storage & Solutions East Germany—which consist of more than 130 partners including universities, industry, and especially SMEs from East Germany. HYPOS, endowed with up to 45 million Euros, focuses on an integrated approach for energy storage, based on renewable energy sources. The major aims of the concept are the provision of green hydrogen in industrial scale and the storage of it in the already existing infrastructures to prove the base load capability of renewable energy resources.

HYPOS also includes a project part dedicated to Power-to-Gas, where the sunfire GmbH has taken the lead role. Within this part, water electrolysis, followed by methanation, is investigated to produce substitute natural gas (SNG). In the course of 2015, after having finished the strategic conceptual phase, the projects of the HYPOS consortium entered the R&D phase.

### Energy storage funding initiative

The Federal Ministries of Economic Affairs and Energy (BMWi) and of Education and Research (BMBF) launched in 2011 a joint initiative to support research in energy storage technologies as part of the “6th Energy Research Program of the Federal Government” (BMWi 2011). The desire for a balanced electricity grid and for a vital expansion of renewable energy raises the need of short- and long-term energy storage as described above. Since 2012, both ministries, BMWi and BMBF, granted for R&D on energy storage technologies almost 200 million Euros for more than 280 projects. Ten of the collaborative projects are related to the topic of “chemical energy storage using CO_2_” which are mostly work-in processes.

The Siemens-led joint project “Li-Kohle” (lithium coal) worked on a closed energy storage cycle, based on lithium. With excess renewable energy, lithium will be recycled from combustion residues. In times of an energy shortage, lithium can be combusted in the presence of CO_2_ or nitrogen. Besides, the operational reserve for the energy grid, very attractive side products, such as carbon monoxide, methanol, or ammonia, results from this process (Schiemann et al. 2014). The project finished just recently on the TRL 3–4.

In the project “Katmethan,” researchers investigate peptide-based catalysts for the economic synthesis of methane from renewable energy, CO_2_, and water. The project has only recently started (TRL 1–2). In August 2017, the final results are expected.

### Bioeconomy

Other than by chemical means, CO_2_ can also successfully be transformed into value-added products by biotechnological means, such as growing aquatic organisms (algae, cyanobacteria) or land plants. Nowadays, the selection of suitable biomass feedstock from land plants should consider the avoidance of competition with nutrition (table vs. tank). Biotechnology offers various options alongside the value chain to make new products from CO_2_ accessible or to improve the efficiency, e.g., in agricultural or industrial processes, respectively. Since 2010, when the BMBF launched the “National Research Strategy Bioeconomy 2030,” NFS 2030 (BMBF 2010), the emphasis on industrial biotechnology was strengthened. Two of the five key fields of action deal with

- Using renewable resources for the industry
- Developing biomass-based energy carriers

and thus relate to the biotechnological utilization of CO_2_. The “Innovation-Initiative Industrial Biotechnology” (BMBF 2011b) fosters the formation of intersectoral alliances to replace fossil resources based on biotechnological products in order to switch to renewable resources and reduce energy consumption. One hundred million Euros were reserved for funding the alliances. The first five collaboration teams were selected recently, but more may follow soon (Müller 2014). Three of the five selected alliances are dedicated to CCU technologies: (I) “ZeroCarbFP—functional biomass from carbon rich waste streams,” (II) “Technofunctional proteins—TeFuProt,” and (III) “Functionalization of proteins—FuPol.” The BMBF supports the three teams with 32.5 million Euros of public support, most of it, 24 million Euros, allocated to ZeroCarbFP.

Within the “Biotechnology 2020+” strategy process (BMBF 2015e), the funding measure “Basic technologies for a next generation of biotechnological processes” (BMBF 2011a) supports three selected projects with regards to microbial or enzymatic activation of CO_2_ with a total budget of 4.3 million Euros: The first analysis and designs bacterial enzyme cascades for the use of CO_2_. The second focuses on bioelectrosynthesis for the production of materials from CO_2_. The development of enzymatic-chemocatalytic oxidation cascades in the gas phase is issued by the third project team. Even though these projects are still positioned in basic research, in the future, biotechnological approaches will play a major role in CO_2_ conversion.

Former BMBF funding measures of the biotechnology research lead to impressive successes: In July 2015, the consortium of the collaborative project “Advanced Biomass Value,” with Airbus involved among others, announced that the selected algae and oil yeast cultures convert CO_2_ into aviation fuels, lubricants, and construction materials in a more than sufficient way. To cultivate these organisms in an industrial relevant scale, a plate-photo bioreactor took up operation in August 2015 (TRL 6–7). The biomass yield per area is expected to exceed rooted plants by the factor of 10–100 (IBB 2015).

### Impulses for Industrial Resource Efficiency

Numerous innovative approaches have been investigated and developed in public-funded projects with respect to resource efficiency. The gap between research and industrial application, we call it the “valley of hope,” is for many of those promising projects an insuperable obstacle. Especially in the field of raw material efficiency, which includes the extension of the raw material base via CCU, the valley of hope is often recognized as “valley of death.” To follow up on most promising results of the raw material R&D projects and to overcome obstacles that block the way into the market, BMBF has launched a novel innovation funding instrument: “r + Impuls—Innovative Technologies for Resource Efficiency—Impulses for Industrial Resource Efficiency.” By funding the accompanying research of a scale-up process, BMBF aims to cushion the high risks associated with such a transfer. Industrial lead consortia submitted proposals until March 1, 2016 (BMBF 2014a).

### CCU to broaden the (carbon) raw material base

The projects of the funding measure “Technologies for Sustainability and Climate Protection: Chemical Processes and Use of CO_2_” are coming to a close. As stated in this article before, outstanding results underlined the importance of the research field and led to unexpected discoveries and successes. Based on this funding measure, BMBF designed and published the call: “CO_2_Plus—Broadening the Raw Material Base by CO_2_ Utilization” in June 2015 (BMBF 2015a).

One major aim of the call is to provide support for further need of research as identified by the current funding measure. Therefore, the integration of CO_2_ into value-added chains, i.e., polymers and C1-based chemicals, are addressed by this funding measure. Another focus lies on the intensification of research of previously under-represented areas that show great potential with respect to high-tech innovations, including photo- and electrocatalysis, or direct air capture of CO_2_. Special attention will be paid to cross-industry approaches, integrating the chemical industry and the process industry, e.g., steel and cement. The call closed in October 2015 and the selected projects are planned to start in the second half of 2016.

### Kopernikus Power-to-X Project for the Energy Transition

Most recently, in September 2015, BMBF published the call for proposals “Kopernikus Projects for the Energy Transition,” which is endowed with up to 10 million Euros p.a. for a Power-to-X project until 2025 (BMBF 2015b; BMBF 2015c). This initiative is part of afore mentioned “6th Energy Research Programme of the Federal Government.” The aim of this call is to identify relevant technologies for the transition of the energy system and to develop the selected technologies to large-scale applications. The question, how flexible excess energy from renewables could be used or stored profitably, will be addressed by the project “Flexible use of renewable resources: Power-to-X.” Within the project, Power-to-X technologies are developed to a commercial scale to provide means of utilizing more than 90% of the excess renewable energy by 2026. The focus lies on R&D of processes to use renewable electricity for the generation of chemical energy carriers, for instance hydrogen or methane (Power-to-Gas), liquid fuels (Power-to-Liquid), and base chemicals for the processing industry (Power-to-Chem). Thus, the utilization of CO_2_ plays a major role in this respective Kopernikus project (BMBF 2015b).

## Concluding remarks

The transition of the raw material base towards an almost carbon neutral, circular economy is one of the biggest challenges on the way to a green economy. CO_2_ utilization technologies can deliver solutions to changing and broadening the raw material base and contribute to reduce greenhouse gas emissions. To foster innovation, Germany supports a broad variety of CCU-related R&D projects, from fundamental research to industrial relevant demonstration plants, from catalysis and biotechnology to processing and engineering, from clean fuels to CO_2_-based chemicals and subsequent products. As the utilization of CO_2_ as a raw material becomes profitable for companies, the dependency of the chemical industry and the mobility sector on fossil carbon sources will decrease. The majority of new CCU-technologies are not commercially viable today. But because the United Nations Climate Change Conference in Paris (COP 21, November/December 2015) set global ambitious CO_2_-reduction targets and measures, e.g., for the mobility sector, CCU technologies can contribute significantly moving towards a green economy.
